# Oral vinorelbine versus intravenous vinorelbine, in combination with epirubicin as first-line chemotherapy in Chinese patients with metastatic breast cancer

**DOI:** 10.1007/s00280-019-04000-3

**Published:** 2019-12-14

**Authors:** Liang Huang, Xiaojia Wang, Liheng Zhou, Lijun Di, Hongyu Zheng, Zefei Jiang, Yongsheng Wang, Xiangqun Song, Jifeng Feng, Shiying Yu, Yunpeng Liu, Hong Zheng, Kunwei Shen, Zhongsheng Tong, Zhimin Shao

**Affiliations:** 1 Department of Breast Surgery, Fudan University Shanghai Cancer Center/Cancer Institute, 399 Ling-Ling Road, Shanghai, 200032 People’s Republic of China; 2 Zhejiang Cancer Hospital, No.1, East Banshan Road, Gongshu District, Hangzhou, 310022 People’s Republic of China; 3 Shanghai Jiatong University School of Medicine Renji Hospital, 145 Shandong Middle Rd, Huangpu Qu, Shanghai, 200333 People’s Republic of China; 4 Beijing Cancer Hospital, 52 Fucheng Rd, Wu Ke Song, Beijing, 100091 People’s Republic of China; 5 Fujian Provincial Cancer Hospital, 91 Fengban Maluding, Fuma Lu, Jin’an District, Fuzhou, 350014 People’s Republic of China; 6 307 Hospital of PLA, 8 East St, Fengtai Qu, Beijing, 100160 People’s Republic of China; 7 Shangdong Cancer Hospital, Jinan, 250117 People’s Republic of China; 8 Guangxi Medical University Affiliated Cancer Hospital, Fukang Rd, Qingxiu Qu, Nanning, 530015 People’s Republic of China; 9 Jiangsu Cancer Hospital, Nanjing, 210009 Jiangsu People’s Republic of China; 10 Tongji Hospital, No.1095 Jie Fang Avenue, Hankou, Wuhan, 430030 People’s Republic of China; 11 The First Hospital of China Medical University, Taiyuan Street Business Area, Heping, Shenyang, 110003 People’s Republic of China; 12 West China Hospital of Sichuan University, No.37 Guoxue Alley, Wuhou District, Chengdu, Sichuan People’s Republic of China; 13 Ruijin Hospital Shanghai Jiao Tong University School of Medicine, No. 197, Rui Jin Er Road, Shanghai, 200025 People’s Republic of China; 14 Tianjin Medical University Cancer Institute and Hospital, Binshui Rd, Hexi Qu, Tianjin, 300011 People’s Republic of China; 15 Department of Oncology, Shanghai Medical College, Fudan University, 138 Yixueyuan Rd, Shanghai, 200333 People’s Republic of China

**Keywords:** Metastatic breast cancer, Oral vinorelbine, Intravenous vinorelbine, Epirubicin, Chinese patients

## Abstract

Oral VRL offers easier administration, better quality of life, and cost saving. This study aimed to evaluate the treatment efficacy in terms of tumor response of the two formulations of vinorelbine (VRL, oral and IV) in combination with epirubicin (EPI); and the effect of EPI co-administration on VRL pharmacokinetics (PK) in Chinese patients with metastatic breast cancer (MBC) using a phase 2, open label, randomized trial. Patients were aged 18–70 years, had histologically confirmed MBC, Karnofsky Performance Status ≥ 70%, and life expectancy ≥ 12 weeks. The treatment consisted of 6 cycles of 3 weeks each. VRL dose was: (Oral-VRL) 60 mg/m^2^ for cycle 1, 80 mg/m^2^ for cycles 2–6, and (IV-VRL) 25 mg/m^2^ for cycle 1 and 30 mg/m^2^ for cycles 2–6. EPI dose of 75 mg/m^2^ was given on day 1 in both arms for all cycles. 133 patients were enrolled: 66 in Oral-VRL and 67 in IV-VRL arms. The median age for Oral-VRL and IV-VRL arms was 48.4 and 50.0 years, respectively. Objective response rates were 50.0% (95% CI 37.4–62.6%) for Oral-VRL and 53.7% (95% CI 41.1–66.0%) for IV-VRL. Both treatment arms met the efficacy objective target of at least 31 responses, demonstrating efficacy as first-line treatment for MBC. Similar blood PK profiles, exposures, and VRL clearance were observed between VRL + EPI vs VRL-only modalities for both arms. Oral VRL is comparable to IV VRL and an effective first-line treatment for Chinese patients with MBC. The activity of VRL + EPI combination is unaltered when VRL is given orally at recommended doses.

## Introduction

In China, breast cancer is the most common cancer in women, especially among those aged between 30 and 59 years and in urban areas, which had twice the incidence rate compared with rural areas [1, 2]. An estimated 1.6 million people in the country were diagnosed with breast cancer in 2014 and 1.2 million people succumbed to the disease annually [3]. This accounted for 12.2% of newly diagnosed breast cancers and 9.6% of deaths from breast cancer worldwide, and the incidence of breast cancer continues to rise by 1.1% annually [1].

Despite adjuvant chemotherapy, 25–30% of patients without, and 75–80% with histological axillary node involvement were expected to have recurrent and/or metastatic breast cancer (MBC) within 10 years and eventually succumb to the disease [4]. For the majority of patients with MBC, the disease is incurable, and the main treatment goal for the patient is palliation, with the aim of maintaining or improving the quality of life and possibly, duration of survival [5]. Nevertheless, incremental improvements in the duration of survival (estimated at ~ 20 months, range 13.2–29.5 months) have been achieved in the first-line treatment of advanced breast cancer, coincident with the use of new therapies and augmented with supportive care and improved diagnostic techniques [6].

Given its good therapeutic index, hormonal therapy is considered the first option for women with estrogen receptor or progesterone receptor positive disease with minor visceral involvement [7]. However, endocrine resistance typically occurs during the course of the disease and for most patients, cytotoxic chemotherapy is the mainstream treatment for MBC [8]. It offers disease control and palliative benefits, and improves survival and quality of life [9], especially in patients with hormonally insensitive disease or for whom hormonal therapies have failed.

Combination chemotherapy is the most commonly used palliative treatment for MBC, and the main components of many standard regimens are taxanes and/or anthracyclines [10]. Chemotherapeutic agents with original mechanisms of action such as vinorelbine (VRL, Navelbine^®^, Pierre Fabre Oncologie, Boulogne, France), capecitabine, and eribulin can provide further options for first-line treatment [8]. Vinorelbine, a 3rd generation vinca alkaloid, has shown a high therapeutic index compared to other vinca alkaloids. It is associated with lower neurotoxicity [11] and has been shown to be effective and well-tolerated in the treatment of MBC [12–14]. VRL has already been used in combination with anthracyclines in several clinical trials [15–17]. Clinical experience on the combination of intravenous (IV) VRL and epirubicin (EPI) for first-line treatment of metastatic breast cancer is rather extensive [16–18]. The oral formulation of vinorelbine is available as gelatine capsules. This drug formulation resolved earlier issues regarding drug stability and absorption of the active drug and its excipient [11, 19]. This oral formulation has several advantages over the IV form: (1) it is easier to administer, (2) improves the quality of life in the palliative setting, and (3) lowers the cost of medical care as it avoids hospitalization and reduces administration cost [20, 21]. Oral VRL is thus a useful alternative to the IV form and deserves further clinical investigation.

Studies on the use of oral VRL as a single-agent [22, 23] or in combination therapy with EPI [24–26] for the treatment of MBC in Asian patients were limited. The aim of this study was to evaluate the treatment efficacy in terms of tumor response of the two formulations of VRL (oral and IV), when used in combination with standard dose EPI, and the effect of EPI co-administration on VRL pharmacokinetics (PK) in Chinese patients with MBC.

## Materials and methods

This phase 2, prospective, open label, multi-center, randomised trial enrolled Chinese patients with metastatic breast cancer between February 2008 and January 2010 at 12 sites in China. The protocol was approved by the Independent Ethics Committees at each site prior to the start of the study. The study was conducted in accordance with the ethical principles stated in the Declaration of Helsinki and subsequent amendments, and in compliance with Good Clinical Practice Guidelines (Committee for Proprietary Medicinal Products (CPMP)/International Conference on Harmonization (ICH/135/95). All patients signed informed consent prior to entry into the study.

### Patients

Eligible patients were aged between 18 and 70 years, both inclusive. Those aged > 65 years must not have > 3 comorbidities which impacted cardiac, pulmonary, liver or renal functions. Patients must have (1) histologically confirmed adenocarcinoma of the breast and metastatic disease previously untreated by chemotherapy; (2) Karnofsky Performance Status (PS) ≥ 70%; and (3) a life expectancy ≥ 12 weeks. Prior adjuvant or neoadjuvant chemotherapy which contained an anthracycline and/or taxane (maximum cumulative dose: 360 mg/m^2^ for doxorubicin, 540 mg/m^2^ for EPI) and relapsing > 6 months after the end of adjuvant chemotherapy, or prior hormonal therapy for metastatic breast cancer, was allowed. Patients may receive prior radiotherapy but not on sites used to assess response and where a minimum of 4 weeks’ interval have elapsed. They had adequate bone marrow, hepatic, and renal functions, normal cardiac function, presence of at least one measurable indicator lesion (RECIST 1.0 criteria [27]) which was not previously irradiated, LVEF ≥ 50% as measured by MUGA scan or ultrasound, and absence of psychological, familial, sociological or geographical conditions potentially hampering compliance with the study protocol and follow-up schedule.

Patients with poor disease prognosis such as inflammatory (T4d) disease, bilateral cancer, symptomatic lung lymphangitis; prior chemotherapy in the metastatic setting; concomitant hormone therapy for metastatic breast cancer, or previously treated with a vinca-alkaloid but relapsing < 6 months after the chemotherapy were excluded. Other exclusion criteria were: active central nervous disorder, brain metastasis or leptomeningeal involvement; symptomatic neuropathy (sensory) > grade 1 according to the National Cancer Institute Common Toxicity Criteria (NCI–CTC V2) [28]; concomitant/uncontrolled medical disorder (cardiac failure or myocardial infarction within the previous 3 months, uncontrolled hypertension or arrhythmia, unstable diabetes, uncontrolled hypercalcaemia, and clinically significant active infection requiring IV antibiotics within 2 weeks before the beginning of treatment).

### Study design

Patients were randomized (1:1) into two arms: oral VRL + EPI (Oral-VRL arm, 60 patients) or IV VRL + EPI (IV-VRL arm, 60 patients). The study drug administration was initiated within 7 days after randomisation. The treatment consisted of 6 cycles of 3 weeks each. For each cycle, VRL was given on days 1 and 8 of the cycle. The administered dose of VRL for the Oral-VRL arm was 60 mg/m^2^ for cycle 1 and 80 mg/m^2^ for cycles 2–6 with a maximum dose of 120 and 160 mg, respectively. For the IV-VRL arm, the VRL dose was 25 mg/m^2^ for cycle 1 and 30 mg/m^2^ for cycles 2–6 with maximum doses of 50 and 60 mg, respectively. EPI was infused at the dose of 75 mg/m^2^ on day 1 in both arms for all cycles. Drug administration was cancelled in the event of disease progression, unacceptable toxicity or at patient’s refusal. A patient was withdrawn if a cycle lasted > 5 weeks (i.e. > 2 weeks delay). Preventive antiemetic was given on day 1 according to the institutional protocols for each EPI/VRL administration and systematic antiemetic treatment with oral 5-HT3 antagonist, on day 8 prior to oral VRL administration.

### Study procedures

Physical examination including vital signs, body weight and performance status was performed on day 1 of each cycle. Complete blood cell and platelet counts were performed on day 1 and day 8, and serum biochemistry on day 1 of each cycle. Each patient was followed for 30 days after the last drug administration and survival information was collected every 3 months.

Blood sampling for the VRL PK and its active metabolite 4-O-deacetylvinorelbine (DVRL) was performed for both arms in cycle 1 according to a six time-points limited sampling schedule after the co-administration of VRL and EPI on day 1 and administration of VRL alone on day 8. VRL and DVRL were assayed in whole blood by LC/MS–MS with a LLOQ of 0.25 ng/mL.

### Study assessments

The primary efficacy analysis was to assess the Objective Response Rate (ORR), i.e. patients with complete or partial remission, in both arms on the ITT population. Tumour assessment was performed according to the RECIST 1.0 method [27] at baseline and after every two cycles. After the completion of six cycles, the assessment was performed every 3 months until tumour progression was documented. Confirmation of an objective response was performed at least 4 weeks after documentation. A minimum of 31 responses was required to meet the efficacy objective. The secondary efficacy parameters analysed were: disease control rate, time to first response, duration of response, time to treatment failure, progression-free survival and overall survival. All adverse events were reported using the NCI-CTC system [28]. Maximum grade or severity was reported by cycle and by patient.

A subset of patients who completed their first cycle of treatment and had their blood sampled on days 1 and 8 of that cycle were evaluated for the effect of EPI co-administration on VRL PK. For each route of administration, the EPI drug–drug interaction analysis was evaluated by comparing Bayesian VRL PK parameters between day 1 (VRL + EPI) and day 8 (VRL alone).

### Statistical analyses

The sample size was calculated according to the Fleming method [29] using reference response rates (po = 0.40, pA = 0.60), error probabilities (*α* ≤ 0.05, *β* ≤ 0.10) and number of tests (*k* = 2). Assuming a 10% non-evaluable rate, 132 patients were assigned for the trial. All descriptive statistics are presented in summary tables.

The analysis was performed on the intent-to-treat (ITT) population. All treated patients were included in the safety analysis unless the patient was lost to follow-up immediately after the start of the treatment. Continuous data are summarized with frequency and median [range]. Categorical data are presented in contingency tables with frequencies and percentages. The tumour response rate was assessed and 95% CI were calculated following the exact method. Analyses of disease control rate, time to first response, duration of response, duration of disease control, progression-free survival and time to treatment failure was also performed, while overall survival was analysed with the Kaplan–Meier method. Statistical evaluation of drug bioavailability between days 1 and 8 was performed by a 2-way analysis of variance (5% nominal *α* risk). Statistical analyses were carried out with SAS^**®**^ version 8.2 for Windows^**®**^ (SAS Institute Inc., 100 SAS Campus Drive Cary, NC 27513-2414, USA).

## Results

### Patients

**Fig. 1:**
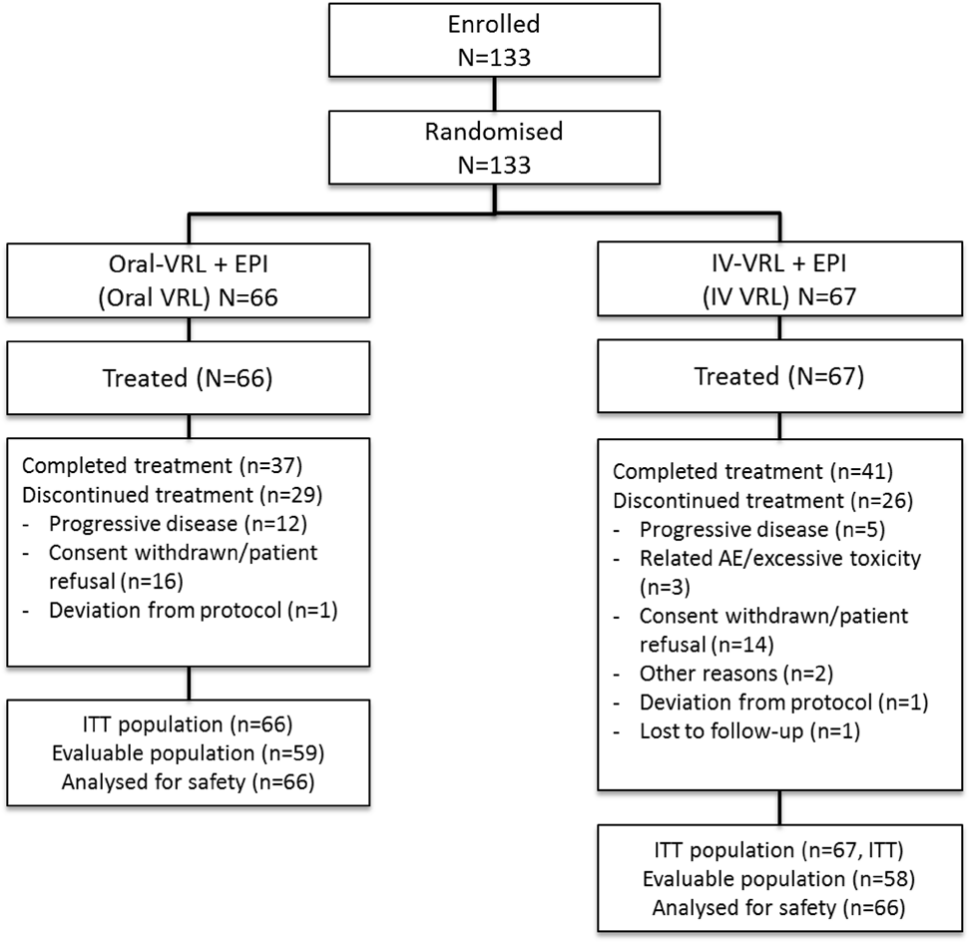
Patient disposition. *AE* adverse event, *EPI* epirubicin, *ITT* intent-to-treat, *IV* intravenous, *VRL* vinorelbine

Of the 133 patients enrolled, 66 patients were randomized to Oral-VRL and 67 to IV-VRL arms (Fig. 1). The number of patients who completed the treatment as per protocol in the Oral-VRL and IV-VRL arms was 37 and 41, respectively. As at the end of study (Feb 2011), 55 patients (Oral-VRL: 29, IV-VRL: 26) had discontinued treatment and the main reason was patient refusal or withdrawal of consent (Oral-VRL: 16, IV-VRL: 14).

The median age of patients in Oral-VRL and IV-VRL arms was 48.4 (32.2–68.8) and 50.0 (31.3–65.7) years, respectively. Table 1 shows the patient demographics and disease characteristics. The two arms were similar in their age distribution profiles. The Oral-VRL arm had more patients at 70% Karnofsky PS: 7 (10.6%) vs 1 (1.5%) in IV-VRL arm, and less patients at 90%: 44 (66.7%) vs 51 (76.1%) in IV-VRL arm. At diagnosis, most patients had ductal (Oral-VRL: 53.0%, IV-VRL: 64.2%) or invasive (Oral-VRL: 22.7%, IV-VRL: 13.4%) cancers. At study entry, there were 17 patients (12.8%) at stage IV, with more patients in Oral-VRL arm (10 patients, 15.2%) compared to IV-VRL arm (7 patients, 10.4%). More patients in the Oral-VRL arm had ≥ 3 organs involved (30 patients, 45.5% vs 20 patients, 29.9% patients in IV-VRL arm); and liver metastases (23 patients, 34.8% vs 18 patients, 26.9% in IV-VRL arm).

**Table 1:** Patient demographics and disease characteristics (ITT population)

	Oral-VRL arm		IV-VRL arm		All	
	*N* = 66	100	*N* = 67	100	*N* = 133	100
	*n*	%	*n*	%	*n*	%
Age distribution, years						
< 35	2	3.0	4	6.0	6	4.5
35 to < 50	34	51.5	30	44.8	64	48.1
50 to < 65	29	43.9	32	47.8	61	45.9
≥ 65	1	1.5	1	1.5	2	1.5
Karnofsky PS						
70	7	10.6	1	1.5	8	6.0
80	8	12.1	9	13.4	17	12.8
90	44	66.7	51	76.1	95	71.4
100	7	10.6	6	9.0	13	9.8
At diagnosis						
Histology						
Ductal, NOS	35	53.0	43	64.2	78	58.6
Invasive, NOS	15	22.7	9	13.4	24	18.0
Intraductal	4	6.1	1	1.5	5	3.8
Invasive with predominant intraductal component	4	6.1	4	6.0	8	6.0
Cancer, NOS	3	4.5	3	4.5	6	4.5
Invasive	2	3.0	2	3.0	4	3.0
Medullary with lymphocytic infiltrate	1	1.5	–	–	1	0.8
Lobular	–	–	2	3.0	2	1.5
Mucinous	–	–	2	3.0	2	1.5
Unknown	2	3.0	1	1.5	3	2.3
Hormonal receptor status (Oestrogen/Proesterone)						
Negative/negative	17	25.8	21	31.3	38	28.6
Negative/positive	5	7.6	4	6.0	9	6.8
Positive/negative	9	13.6	10	14.9	19	14.3
Positive/positive	26	39.4	26	38.8	52	39.1
Unknown/negative	1	1.5	1	1.5	2	1.5
Unknown/unknown	8	12.1	5	7.5	13	9.8
At study entry						
Stage						
Relapse	56	84.8	60	89.7	116	87.2
Stage IV	10	15.2	7	10.4	7	12.8
Number of organs involved						
1 organ	16	24.2	23	34.3	39	29.3
2 organs	20	30.3	24	35.8	44	33.1
3 or more organs	30	45.5	20	29.9	50	37.1

*EPI* epirubicin, *ITT* intent-to-treat, *NOS* not otherwise specified, *PS* performance status, *VRL* vinorelbine

### Treatment exposure

The median treatment duration for Oral-VRL and IV-VRL arms was 18.5 (3.0–23.9) and 18.7 (3.0–22.7) weeks, during which, patients in both arms had a median of 6 (1–6) cycles of treatment. The median dose intensity (DI) for VRL was 40.0 mg/m^2^/week in Oral-VRL arm and 15.4 mg/m^2^/week for in IV-VRL arm. The relative dose intensity (RDI) was 80.0% and 80.3%, respectively. For EPI, the median DI was 22.2 mg/m^2^/week in Oral-VRL arm and 21.8 mg/m^2^/week in IV-VRL arm with a RDI of 88.8% and 87.2% for both arms, respectively. The percentage of patients who had their VRL doses reduced were similar in both arms (24.2% vs 22.4%). However, more patients had their EPI doses reduced in IV-VRL arm (31.3% vs 18.2% in Oral-VRL arm). The most common reason for a dose reduction was drug-related haematological toxicity: 14/17 (82.4%) for Oral-VRL, 15/15 (100%) for IV-VRL, and for EPI, 9/12 (75.0%) and 20/21 (95.2%) in Oral-VRL and IV-VRL arms, respectively.

From cycle 2 onwards, the percentage of cycles delayed at day 1 in Oral-VRL and IV-VRL arms was similar, i.e. 69/253 (27.3%) and 74/267 (27.7%), respectively. A higher percentage of patients on IV VRL had their day 8 drug administration delayed (18.1% vs 8.3% for Oral-VRL arm) or cancelled (11.2% vs 7.7% for Oral-VRL arm). The most frequent reason for a cancelled VRL administration was neutropenia, accounting for 33 (89.2%) and 15 (62.5%) of cancellations in IV VRL and Oral-VRL arms, respectively.

Of the 133 patients, 130 (97.7%) advanced to cycle 2, and 71 (53.4%) patients received the escalated VRL doses as planned: 41 (62.1%) and 30 (44.8%) in Oral-VRL and IV-VRL arms, respectively. The other 59 patients received the same doses as in cycle 1: 24 (36.4%) and 35 (52.2%) patients in Oral-VRL arm and IV-VRL arms, respectively. Neutropaenia was the most common reason for non-escalation of doses: 15/24 (62.5%) and 28/35 (80.0%) in Oral-VRL and IV-VRL arms, respectively.

### Treatment efficacy

For the ITT population, both arms achieved similar results for the primary efficacy endpoint, ORR, i.e. 50.0% and 53.7% for Oral-VRL arm and IV-VRL arms, respectively (Table 2). Both arms met the treatment efficacy objective target of a minimum of 31 responses. In Oral-VRL arm, 86.4% (CI 75.7–93.6) of patients had the disease under control and similarly, 88.0% (CI 77.8–94.7) in IV-VRL arm. The median times to first response and to treatment failure in both arms were similar. At the end of the study, the percentages of patients in the Oral-VRL and IV-VRL arms who had died or relapsed were 34.8% (23/66) and 32.8% (22/67), respectively. The median follow-up times for both arms were 7.6 and 8.2 months, respectively. Six patients in each arm deceased during the follow-up period. The progression-free survival and overall survival were not computed as there were insufficient patient data-points.

**Table 2:** Efficacy Endpoints (ITT population)

	Oral-VRL arm (*N* = 66)	IV-VRL arm (*N* = 67)
Number of patients		
Evaluable population^a^, *n* (%)	60 (90.9)	60 (89.6)
Disease under control^b^, *n* (%)	57 (86.4)	59 (88.0)
Partial or complete remission, *n* (%)	33 (50.0)	36 (53.7)
Stable disease, *n* (%)	24 (36.4)	23 (34.3)
Progressive disease, *n* (%)	3 (4.5)	1 (1.5)
Not evaluable, *n* (%)	6 (9.1)	7 (10.4)
Primary endpoint		
Objective response rate (ORR)^c^, %, (95% CI)	50.0 (37.4–62.6)	53.7 (41.6–66.0)
Secondary endpoints:		
Tumor response rate^d^, %	55.0	60.0
Disease under control, % (95% CI)	86.4 (75.7–93.6)	88.0 (77.8–94.7)
Median time to first response (95% CI), months	1.6 (1.3–3.6)	1.8 (1.3–4.9)
Median time to treatment failure (95% CI), months	4.5 (3.7–5.0)	4.6 (4.2–5.0)

^a^Evaluable population: patients evaluable for tumour response were defined as follows: (a) patients who remained on study until the first evaluation and who were evaluated; (b) patients who progressed before the first evaluation were considered as early progression; (c) patients who died from malignant disease before the first evaluation were considered as early death; and (d) patients with baseline lesions assessed at least once after the first cycle, with the same method of measurement as baseline

^b^Disease under control: patients with complete or partial remission or stable disease

^c^Objective response rate: patients who had complete or partial remission of the disease

^d^Tumour response rate: patients who had complete or partial remission of the disease in the evaluable population

### Safety and tolerability

In Oral-VRL arm, 65 patients and 314 cycles were evaluable for the haematological toxicity during therapy administration, and 66 patients and 331 cycles in IV-VRL arm. About 80.0% and 92.4% of patients in Oral-VRL and IV-VRL arms, respectively, had neutropaenia toxicity grades 3/4, with a higher percentage of grade 4 in IV-VRL arm (83.3% vs 58.5% in Oral-VRL arm). These adverse events occurred in 38.2% and 53.1% of cycles in Oral-VRL and IV-VRL arms, respectively. Table 3 shows the number of patients with grades 3/4 toxicity adverse events. Febrile neutropaenia was reported in 9/65 (13.8%) and 14/66 (21.2%) of patients in Oral-VRL and IV-VRL arms and 9/314 (2.8%) and 16/331 (4.8%) of cycles, respectively.

**Table 3:** Adverse events of NCI-CTC toxicity grade 3/4

	Oral-VRL arm			IV-VRL arm		
	All	Grade 3	Grade 4	All	Grade 3	Grade 4
Number of patients	*N* = 65			*N* = 66		
Hematological toxicities, *n* (%)						
Hemoglobin	64 (97.0)	12 (18.5)	–	64 (95.5)	20 (30.3)	5 (7.6)
Leucocytes	61 (92.4)	29 (44.6)	13 (20.0)	66 (98.5)	29 (43.9)	28 (42.4)
Neutrophils	61 (92.4)	14 (21.5)	38 (58.5)	65 (97.0)	6 (9.1)	55 (83.3)
Platelets	55 (83.3)	3 (4.6)	1 (1.5)	56 (83.6)	7 (10.6)	–
Number of patients	*N* = 66			*N* = 67		
Non-haematological toxicities, *n* (%)						
Gastrointestinal disorders						
Nausea	50 (75.8)	8 (12.1)	–	50 (74.6)	10 (14.9)	–
Vomiting	46 (69.7)	14 (21.2)	1 (1.5)	40 (59.7)	9 (13.4)	–
Abdominal Pain	6 (9.1)	–	–	8 (11.9)	2 (3.0)	–
Constipation	9 (13.6)	–	–	12 (17.9)	1 (1.5)	–
Diarrhoea	16 (24.2)	–	–	10 (14.9)	–	1 (1.5)
Stomatitis	7 (10.6)	2 (3.0)	–	8 (11.9)	2 (3.0)	–
General disorders and administration site condition						
Fatigue	41 (62.1)	3 (4.5)	–	38 (56.7)	5 (7.5)	1 (1.5)
Influenza like illness	–	–	–	4 (6.0)		
Pyrexia	8 (12.1)	–	–	16 (23.9)	–	–
Metabolism and nutrition disorders—anorexia	34 (51.5)	4 (6.1)	–	28 (41.8)	5 (7.5)	1 (1.5)
Respiratory, thoracic an mediastinal disorders						
Cough	5 (7.6)	–	–	7 (10.4)	–	–
Interstitial lung disease	–	–	–	4 (6.0)	–	–
Skin and subcutaneous tissue disorders—alopecia	21 (31.8)	–	–	25 (37.3)	–	–
Other investigations						
Weight decreased	16 (24.2)	–	–	15 (22.4)	1 (1.5)	–

*NCI*-*CTC* National cancer institute-common toxicity criteria

The most commonly reported non-haematological adverse events in Oral-VRL arm were nausea (75.8%), vomiting (69.7%) and anorexia (51.5%); and similarly for IV-VRL arm, nausea (74.6%), vomiting (59.7%) and fatigue (56.7%) (Table 3). No patients died within 30 days after the last drug administration. Six patients in Oral-VRL arm and five patients in IV-VRL arm subsequently died of progressive disease. One patient in IV-VRL arm died of pulmonary embolism.

### Pharmacokinetic analysis

**Fig. 2:**
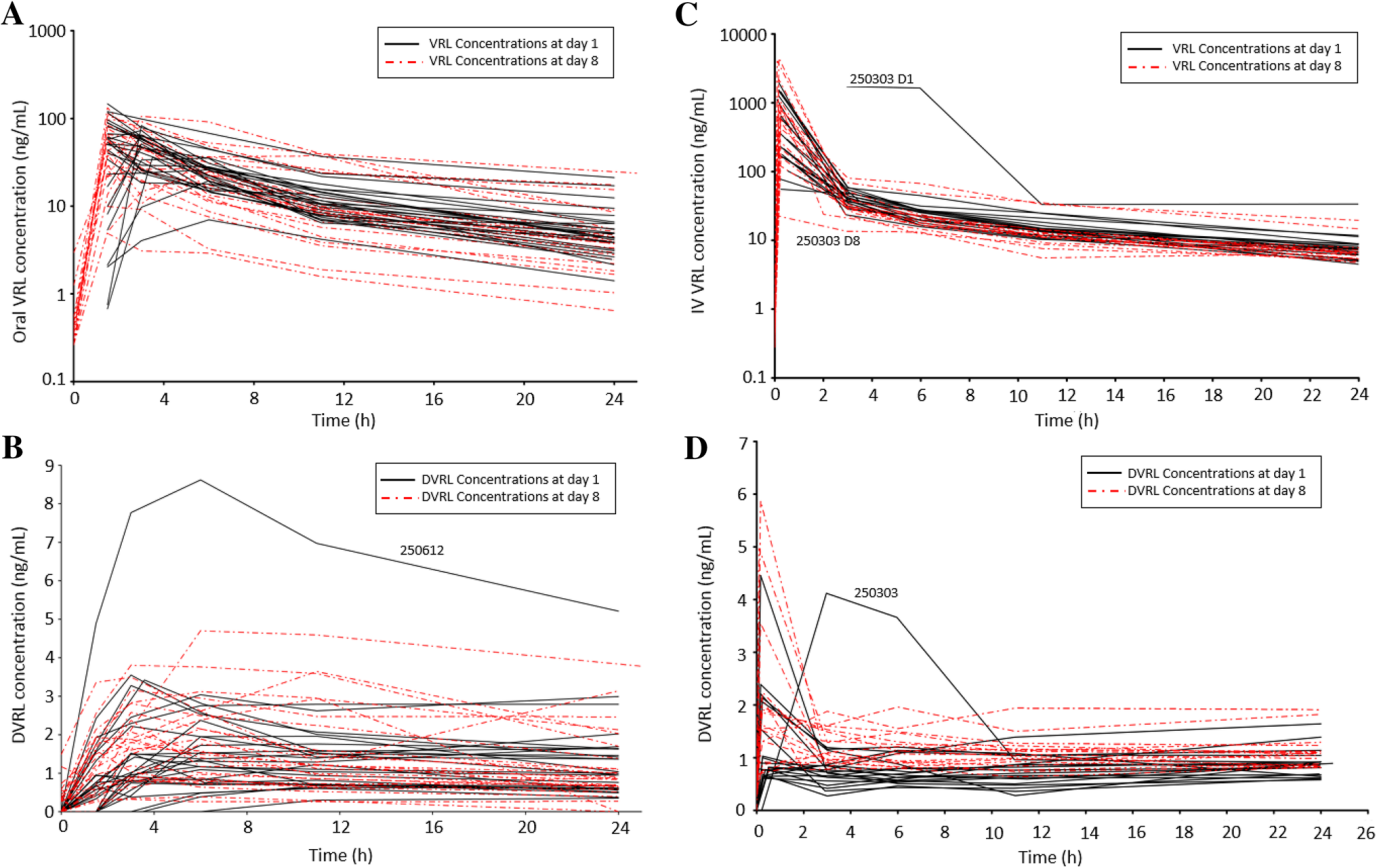
Day 1 and 8 profiles of blood concentrations versus time profiles for **a** oral VRL and **b** its metabolite, DVRL and **c** IV VRL and **d** its metabolite, DVRL. Oral VRL dose was 60 mg/m^2^, IV VRL dose was 25 mg/m^2^. *DVRL* 4-*O*-deacetylvinorelbine, *IV* intravenous, *VRL* vinorelbine

**Fig. 3:**
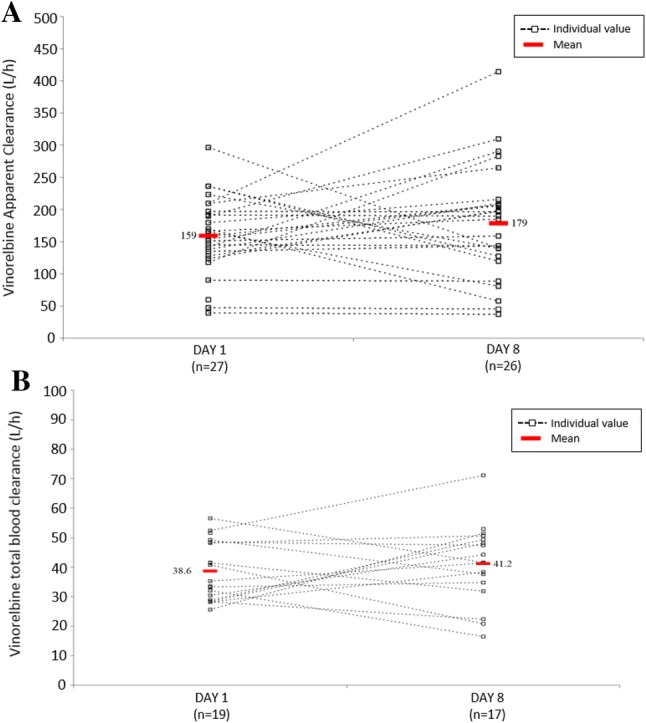
Individual patient profiles for day 1 (VRL + EPI) and day 8 (VRL alone) for **a** apparent clearance, oral VRL and **b** total body clearance, IV VRL. Oral VRL dose was 60 mg/m^2^, IV VRL dose was 25 mg/m^2^. *EPI* epirubicin, *IV* intravenous, *VRL* vinorelbine

For Oral-VRL arm, 27/66 subjects (40.9%) had blood sampled on day 1 and 26/66 (39.4%) on day 8, and for IV-VRL arm, 20/67 (29.8%) and 17/67 (25.4%) on days 1 and 8, respectively. Figure 2 shows the individual VRL blood concentration profiles over time for VRL and its metabolite, DVRL for both arms. The day 1 profiles were superimposed over day 8 profiles. For both arms, the day 1 and 8 profiles overlapped and showed no differences in the PK between day 1 (VRL + EPI) and day 8 (VRL) (Figs. 3a, b). For IV-VRL, one patient was excluded as an outlier. The superimposed DVRL blood concentration profiles for days 1 and 8 in both arms were also similar (Figs. 3c, d). The metabolite exhibited low concentration levels (< 10 ng/mL). As in the VRL profile, the same outlier patient was excluded from the analysis.

The individual patient values for apparent clearance (Oral-VRL arm) and total body clearance (IV-VRL arm) at day 1 and day 8 are displayed in Fig. 3. In both arms, there was no obvious trend between the day 1 and day 8 patterns showing that the mean clearance and blood exposures were similar whether VRL was associated with EPI or not.

## Discussion

This phase II study compared the oral and IV formulations of VRL in combination with EPI for the treatment of Chinese patients with MBC. Both therapies achieved similar ORRs: 50.0% (95% CI 37.4–62.6%) for Oral-VRL arm and 53.7% (95% CI 41.1–66.0%) for IV-VRL arm, and surpassed the treatment efficacy target of at least 31 responses. This demonstrated their efficacies as first-line treatments for MBC. The results were also consistent with treatment responses reported in a number of phase II/III studies of VRL as a first-line treatment (38–50% [30–35]) and as a combination therapy with EPI (70.6% [17], 64% [24]) for MBC. Of note is that the efficacy result for the oral VRL in our study was achieved despite the presence of several negative prognostic factors in the Oral-VRL arm: it had more patients who were functionally impaired and had stage IV disease, liver metastases or ≥ 3 organs involved, compared with the IV-VRL arm. The oral combination also delivered similar results for disease control rate, and median times to first response and to treatment failure compared with the IV combination. The median times to first response of 1.6–1.8 months and to treatment failure (4.5–4.6 months) were consistent with the 2 and 10 months, reported by Vici et al. [17] in a phase 2 trial investigating the activity of VRL + EPI as a first-line therapy for MBC.

VRL tolerability for both formulations was similar in our study. There were no differences in the haematological and non-haematological toxicities, an observation which was reported by Bourgeois et al. [36]. In our study, a dose escalation approach had been chosen to optimize the safety profile of the treatment. However, the percentage of patients with adverse events remained on the high side. Haematological toxicities were the most frequent adverse events in both arms, as to be expected from the mechanisms of action of VRL and EPI. However, the oral formulation reported less neutropaenia of grade 3/4 (80.0% vs 92.4%) and less febrile neutropaenia (13.8% vs 21.2%) than for IV VRL. In contrast, leukopenia was reported as the dose-limiting toxicity in other VRL studies involving Asian patients [24, 25]. Non-haematological toxicity was similar between both arms. The most frequently reported gastrointestinal disorders, nausea, vomiting and constipation, occurred at low frequencies for grade 3/4 events in both arms. Such side effects can be managed by standard antiemetic prophylaxis and dietary education.

A phase III trial performed by Ejlertsen, et al. [16] demonstrated that the addition of VRL to EPI induced a significant treatment advantage in terms of response rate and progression-free survival. Our study had a short follow-up period of 8 months which did not provide sufficient time to observe the complete progression-free rate and overall survival. Yan, et al. [25], however, observed a 5-year survival rate of 87.9% in a study of 61 Chinese patients with grade II/III breast cancer treated with IV VRL + EPI. EPI was well tolerated in our patients, without signs of chronic heart failure or LVEF observed in other studies on anthracycline-based chemotherapy [37, 38]. This combination, oral VRL + EPI, for the first-line treatment of the metastatic disease is a useful alternative for those patients previously exposed to adjuvant anthracyclines. Our study also showed that the EPI in the combination therapy has limited effect on VRL PK, as suggested by the similar blood PK profiles, mean blood exposures and mean blood clearance of VRL between the two modalities of treatment (VRL + EPI vs VRL only) for both arms.

For patients with metastatic breast cancer, the oral VRL offers the advantages of an oral treatment, i.e. easier administration and better quality of life from greater convenience and comfort, without sacrificing efficacy and safety [21]. The simpler drug administration can alleviate oncology staff shortages, especially nurses and pharmacists, and provide savings in the cost of medical care. Moreover, oral chemotherapy can help to reduce the anxiety in patients who are afraid of injections [39, 40] and it can be a more appropriate route of administration when venous access is problematic. Many patients prefer oral chemotherapy because it improves their quality of life which is an important goal in a palliative setting [15, 20, 41]. The availability of an effective oral chemotherapy is also advantageous for patients living in remote areas or away from oncology centers and clinics. However, the preference of an oral chemotherapy is conditioned by its efficacy that must be equivalent to the IV formulation in terms of treatment and toxicity: this has been demonstrated for VRL in our study.

## Conclusion

Oral VRL in combination with EPI is an effective first-line treatment for Chinese patients with metastatic breast cancer. The efficacy of oral VRL is comparable to IV VRL with similar safety profiles and the activity of the VRL–EPI combination is unaltered when VRL is given orally at the recommended doses.

## Abbreviations

CI: 95% confidence intervals
CPMP: Committee for proprietary medicinal products
DI: Dose intensity
DVRL: 4-*O*-deacetylvinorelbine
EPI: Epirubicin
ICH: International conference on harmonization
ITT: Intent-to-treat
IV: Intravenous
LLOQ: Lower limit of quantification
LC: Liquid chromatography
LVEF: Left ventricular ejection fraction
MBC: Metastatic breast cancer
MS: Mass spectrometry
MUGA: Multigated acquisition
NCI–CTC: National cancer institute common toxicity criteria
ITT: Intent-to-treat
ORR: Objective response rate
PK: Pharmacokinetics
RDI: Relative dose intensity
RECIST: Response evaluation criteria in solid tumors
VRL: Vinorelbine
